# Supplementary material for: Sero-epidemiological evaluation of malaria transmission in The Gambia before and after mass drug administration
Source: BMC Med. 2020 Nov 13;18:331. doi: 10.1186/s12916-020-01785-6 (PMC7664049; doi:10.1186/s12916-020-01785-6)
Supplement: Supplementary file 1 — Additional file 1: Supplementary methods and tables. Supplementary methods describe the antibody acquisition model used in Figs. 4 and 5. Supplementary tables provide values for sero-prevalence, sero-conversion rates, Area Under the Antibody Acquisition curve (AUC), and logistic regression. [file 12916_2020_1785_MOESM1_ESM.docx]

**Supplementary Methods**

**Antibody acquisition model description**

An Ab acquisition model that assumes constant transmission across all ages can be described as:

$A\left( a \right)= \alpha_{0}+ \frac{\alpha}{r} (1- e^{-r*a}$) (1)

Where $A\left( a \right)$ is the geometric mean MFI at age $a$, $\alpha_{0}$ is the baseline antibody intensity, $\alpha$ is the rate of Ab acquisition, and $r$ is the rate of Ab decay in a given population. The model is derived from the differential equation:

$\frac{dA}{dt}= \alpha\left( a \right)-rA$ (2)

Acquisition and decay rates are estimated by assuming that antibody intensity at a given age, $Y(a)$, is log-normally distributed:

$Y\left( a \right)| \alpha_{0}, \alpha, r, a \sim LogNormal ( log \left( A\left( a \right) \right), \sigma^{2} )$ (3)

Similar to reverse catalytic models used to estimate sero-conversion and sero-reversion rates, Ab acquisition models can be extended to estimate different Ab acquisition rates depending on age, which can be described as:

$A\left( a \right)= \left\{ \begin{aligned} \alpha_{0}+ \frac{\alpha_{2}}{r} \left( 1- e^{-r*a_{c}} \right) :a\leq a_{c} \\ \alpha_{0}+ \frac{\alpha_{2}}{r} \left( 1- e^{-r*a_{c}} \right)+ \frac{\alpha_{1}}{r} \left( 1- e^{-r*(a- a_{c)}} \right)e^{-r*a_{c}} :a> a_{c} \end{aligned} \right.$ (4)

Where $a_{c}$ is the age at which there is a change in Ab acquisition rate, $\alpha_{2}$ is the Ab acquisition rate for individuals aged less than or equal to $a_{c}$, $\alpha_{1}$ is the Ab acquisition rate for individuals aged greater than $a_{c}$, and Ab decay rate $r$ is assumed to be constant across all ages. The best fit model for each antigen and study arm or intervention was chosen based on deviance information criterion (DIC) values.

Some short-term markers (Etramp5.Ag1, GexP19, and HSP40) had very small Ab decay rates with respect age, and models excluding this parameter fit the data better (as assessed by DIC values).

The simplified model can be expressed as follows:

$A\left( a \right)= \alpha_{0}+ \alpha*a$ (5)

$A\left( a \right)= \left\{ \begin{aligned} \alpha_{0}+ \alpha_{2}*a_{c} :a\leq a_{c} \\ \alpha_{0}+ \alpha_{2}{*a}_{c}+ \alpha_{1}\left( a- a_{c} \right) :a> a_{c} \end{aligned} \right.$ (6)

All models were fit using Bayesian Monte Carlo Markov Chain (MCMC) estimation with the *rjags* package in R version 3.3.2.

Area under the Ab acquisition curve (AUC) values were calculated based on the Ab acquisition model fit for each cluster and survey month, with 95% credible intervals based on the distribution of estimated Ab intensity for covariate values sampled in the MCMC. This gives an estimate of the cumulative antibody intensity across all ages in the population. (Note: this is different to the AUC values computed in Chapter 5b, which refers to the Area Under the Receiver Operating Characteristic (ROC) curve to define the sensitivity and specificity of a diagnostic test).

**Supplementary Tables**

**Supplementary Table 1. Change in sero-prevalence by antigen, geographical region, and transmission season**

|  | West Coast Region (WCR) | Upper River Region (URR) | |
| --- | --- | --- | --- |
|  | **July – Dec 2013**  **(start – end malaria season)** | **July – Dec 2013**  **(start – end malaria season)** | **Dec 2013 – Dec 2014**  **(pre- and post-MDA)** |
| Etramp5.Ag1 | 4.8% (4.0, 5.6) | 12.9% (11.4, 14.5) | -3.1% (-5.4, -0.9) |
| GEXP18 | 2.2% (0.9, 3.5) | 6.1% (3.6, 8.7) | 3.0% (-0.2, 6.2) |
| HSP40.Ag1 | 0.8% (0.3, 1.4) | 7.2% (5.8, 8.6) | 1.6% (-0.4, 3.6) |
| Rh2.2030 | 0.4% (0.2, 0.6) | 10.3% (8.8, 11.9) | -3.9% (-5.9, -1.9) |
| EBA175 | -0.5% (-0.7, -0.4) | 5.2% (4.2, 6.4) | -1.9% (-3.2, -0.7) |
| *Pf*MSP1_19_ | 3.1% (2.7, 3.5) | 9.4% (8.4, 10.4) | -3.5% (-4.9, -2.1) |
| *Pf*AMA1 | 0.2% (-0.3, 0.8) | 8.1% (5.7, 10.5) | -4.5% (-7.2, -1.8) |
| *Pf*GLURP.R2 | 2.9% (2.3, 3.6) | 8.7% (6.5, 10.8) | -4.3% (-6.7, -1.8) |

Supplementary Table 2. Sero-conversion rates for *Pf*MSP1_19_, *Pf*AMA1, and *Pf*GLURP.R2 by transmission season and geographical region. Mean estimate of sero-conversion rate is shown with 95% confidence interval in parentheses.

|  | *Pf*MSP1_19_ SCR (95%CI) | *Pf*AMA1 SCR (95%CI) | |
| --- | --- | --- | --- |
|  | **SCR overall** | **SCR overall / SCR children*** | **SCR adults** |
| West Coast Region |  |  |  |
| July 2013 | 0.0063 (0.0034 – 0.0115) | 0.0156 (0.0078 – 0.0234) | 0.0896 (0.0448 – 0.1343) |
| December 2013 | 0.0210 (0.0082 – 0.0536) | 0.0126 (0.0063 – 0.0189) | 0.1082 (0.0541 – 0.1624) |
| Upper River Region |  |  |  |
| July 2013 | 0.0131 (0.0088 – 0.0194) | 0.0616 (0.0511 – 0.0743) |  |
| December 2013 | 0.0535 (0.0337 – 0.0849) | 0.0913 (0.0735 – 0.1135) |  |
| April 2014 | 0.0296 (0.0219 – 0.0401) | 0.0742 (0.0628 – 0.0877) |  |
| Dec 2014 | 0.0333 (0.0239 – 0.0464) | 0.0718 (0.0609 – 0.0846) |  |
|  |  | ***Pf*GLURP.R2 SCR (95%CI)** | |
|  |  | **SCR overall / SCR children*** | **SCR adults** |
| West Coast Region |  |  |  |
| July 2013 |  | 0.0166 (0.0083 – 0.0249) | 0.0456 (0.0228 – 0.0684) |
| December 2013 |  | 0.0197 (0.0098 – 0.0295) | 0.0337 (0.0168 – 0.0505) |
| Upper River Region |  |  |  |
| July 2013 |  | 0.0499 (0.0427 – 0.0584) |  |
| December 2013 |  | 0.0708 (0.0605 – 0.0829) |  |
| April 2014 |  | 0.0645 (0.0563 – 0.0738) |  |
| Dec 2014 |  | 0.0645 (0.0322 – 0.0967) | 0.0932 (0.0466 – 0.1397) |

*For clusters with an age-dependent change in sero-conversion rate for *Pf*AMA1 and *Pf*GLURP.R2, SCR for younger ages is listed on left and older ages listed on right. SCR for younger agers is assumed to reflect current intensity and SCR in adults assumed to reflect historical transmission intensity. No change in transmission for *Pf*MSP1_19_ was significant for any clusters or month. Therefore, all *Pf*MSP1_19_ SCRs are listed are for all ages

**Supplementary Table 3. Etramp5.Ag1 AUC values by geographical region and transmission season**

| All ages | AUC (95% CI) | p-value  July/Dec 2013 WCR | p-value  July/Dec 2013 URR | p-value  Dec 2013 / Apr 2014 URR | p-value  Apr/Dec 2014 URR | | p-value  WCR/URR July 2013 | | p-value  WCR/URR Dec 2013 | |
| --- | --- | --- | --- | --- | --- | --- | --- | --- | --- | --- |
| West Coast Region (WCR) | |  |  |  |  |  | |  | |  |
| July 2013 | 16,347 (14,115 – 19,788) | -- | -- | -- | -- | | -- | | -- | |
| Dec 2013 | 17,275 (13,836 – 23,071) | 0·99 | -- | -- | -- | | -- | | -- | |
| Upper River Region (URR) | |  |  |  |  |  | |  | |  |
| July 2013 | 19,880 (16,561 – 23,717) | -- | -- | -- | -- | | 0·99 | | -- | |
| Dec 2013 | 33,748 (25,273 – 47,808) | -- | 0·23 | -- | -- | | -- | | 0·21 | |
| Apr 2014 | 36,388 (30,287 – 43,789) | -- | -- | 1.00 | -- | | -- | | -- | |
| Dec 2014 | 41,101 (33,958 – 49,822) | -- | -- | -- | 0·86 | | -- | | -- | |
| Ages <15 years only | **AUC (95% CI)** | **p-value**  **July/Dec 2013 WCR** | **p-value**  **July/Dec 2013 URR** | **p-value**  **Dec 2013 / Apr 2014 URR** | **p-value**  **Apr/Dec 2014 URR** | | **p-value**  **WCR/URR July 2013** | | **p-value**  **WCR/URR Dec 2013** | |
| West Coast Region (WCR) | |  |  |  |  |  | |  | |  |
| July 2013 | 1,700 (1,489 – 1,935) | -- | -- | -- | -- | | -- | | -- | |
| Dec 2013 | 1,660 (1,368 – 2,011) | 0·98 | -- | -- | -- | | -- | | -- | |
| Upper River Region (URR) | |  |  |  |  |  | |  | |  |
| July 2013 | 1,782 (1,588 – 2,003) | -- | -- | -- | -- | | 0·998 | | -- | |
| Dec 2013 | 3,331 (2,723 – 4,050) | -- | 0·002 | -- | -- | | -- | | 0·004 | |
| Apr 2014 | 3,027 (2,721 – 3,387) | -- | -- | 1.00 | -- | | -- | | -- | |
| Dec 2014 | 3,810 (3,395 – 4,285) | -- | -- | -- | 0·40 | | -- | | -- | |

Supplementary Table 4. GEXP18 AUC values by geographical region and transmission season

| All ages | AUC (95% CI) | p-value  July/Dec 2013 WCR | p-value  July/Dec 2013 URR | p-value  Dec 2013 / Apr 2014 URR | p-value  Apr/Dec 2014 URR | | p-value  WCR/URR July 2013 | | p-value  WCR/URR Dec 2013 | |
| --- | --- | --- | --- | --- | --- | --- | --- | --- | --- | --- |
| West Coast Region (WCR) | |  |  |  |  |  | |  | |  |
| July 2013 | 34,290 (31,808 – 38,644) | -- | -- | -- | -- | | -- | | -- | |
| Dec 2013 | 34,330 (30,963 – 40,688) | 0.997 | -- | -- | -- | | -- | | -- | |
| Upper River Region (URR) | |  |  |  |  |  | |  | |  |
| July 2013 | 46,292 (40,586 – 57,637) | -- | -- | -- | -- | | 0·30 | | -- | |
| Dec 2013 | 55,433 (46,709 – 69,682) | -- | 0·99 | -- | -- | | -- | | 0·28 | |
| Apr 2014 | 54,702 (45,114 – 68,571) | -- | -- | 0·998 | -- | | -- | | -- | |
| Dec 2014 | 68,759 (62,926 – 79,321) | -- | -- | -- | 0·66 | | -- | | -- | |
| Ages <15 years only | **AUC (95% CI)** | **p-value**  **July/Dec 2013 WCR** | **p-value**  **July/Dec 2013 URR** | **p-value**  **Dec 2013 / Apr 2014 URR** | **p-value**  **Apr/Dec 2014 URR** | | **p-value**  **WCR/URR July 2013** | | **p-value**  **WCR/URR Dec 2013** | |
| West Coast Region (WCR) | |  |  |  |  |  | |  | |  |
| July 2013 | 4,643 (4,286 – 5,017) | -- | -- | -- | -- | | -- | | -- | |
| Dec 2013 | 4,533 (4,079 – 5,030) | 0·998 | -- | -- | -- | | -- | | -- | |
| Upper River Region (URR) | |  |  |  |  |  | |  | |  |
| July 2013 | 6,216 (5,425 – 7,089) | -- | -- | -- | -- | | 0·03 | | -- | |
| Dec 2013 | 6,904 (6,069 – 7,811) | -- | 0.99 | -- | -- | | -- | | 0·007 | |
| Apr 2014 | 6,444 (5,657 – 7,355) | -- | -- | 0·99 | -- | | -- | | -- | |
| Dec 2014 | 9,612 (8,846 – 10,383) | -- | -- | -- | 0·001 | | -- | | -- | |

Supplementary Table 5. HSP40.Ag1 AUC values by geographical region and transmission season

| All ages | AUC (95% CI) | p-value  July/Dec 2013 WCR | p-value  July/Dec 2013 URR | p-value  Dec 2013 / Apr 2014 URR | p-value  Apr/Dec 2014 URR | | p-value  WCR/URR July 2013 | | p-value  WCR/URR Dec 2013 |
| --- | --- | --- | --- | --- | --- | --- | --- | --- | --- |
| West Coast Region (WCR) | |  |  |  |  |  | |  | |
| July 2013 | 23,297 (20,173 – 26,970) | -- | -- | -- | -- | | -- | | -- |
| Dec 2013 | 15,280 (12,841 – 19,253) | 0·11 | -- | -- | -- | | -- | | -- |
| Upper River Region (URR) | |  |  |  |  |  | |  | |
| July 2013 | 33,811 (27,803 – 41,624) | -- | -- | -- | -- | | 0·18 | | -- |
| Dec 2013 | 33,181 (25,953 – 43,285) | -- | 0·996 | -- | -- | | -- | | 0·12 |
| Apr 2014 | 38,409 (28,874 – 50,755) | -- | -- | 0·99 | -- | | -- | | -- |
| Dec 2014 | 49,502 (38,483 – 62,888) | -- | -- | -- | 0·97 | | -- | | -- |
| Ages <15 years only | **AUC (95% CI)** | **p-value**  **July/Dec 2013 WCR** | **p-value**  **July/Dec 2013 URR** | **p-value**  **Dec 2013 / Apr 2014 URR** | **p-value**  **Apr/Dec 2014 URR** | | **p-value**  **WCR/URR July 2013** | | **p-value**  **WCR/URR Dec 2013** |
| West Coast Region (WCR) | |  |  |  |  |  | |  | |
| July 2013 | 2,356 (2,129 – 2,605) | -- | -- | -- | -- | | -- | | -- |
| Dec 2013 | 1,504 (1,291 – 1,757) | 0·01 | -- | -- | -- | | -- | | -- |
| Upper River Region (URR) | |  |  |  |  |  | |  | |
| July 2013 | 3,211 (2,820 – 3,647) | -- | -- | -- | -- | | 0·11 | | -- |
| Dec 2013 | 3,342 (2,850 – 3,913) | -- | 0·995 | -- | -- | | -- | | <0·001 |
| Apr 2014 | 3,179 (2,700 – 3,766) | -- | -- | 0·999 | -- | | -- | | -- |
| Dec 2014 | 3,765 (3,267 – 4,397) | -- | -- | -- | 0·989 | | -- | | -- |

Supplementary Table 6. EBA175 AUC values by geographical region and transmission season

| All ages | AUC (95% CI) | p-value  July/Dec 2013 WCR | p-value  July/Dec 2013 URR | p-value  Dec 2013 / Apr 2014 URR | p-value  Apr/Dec 2014 URR | | p-value  WCR/URR July 2013 | | p-value  WCR/URR Dec 2013 | |
| --- | --- | --- | --- | --- | --- | --- | --- | --- | --- | --- |
| West Coast Region (WCR) | |  |  |  |  |  | |  | |  |
| July 2013 | 37,163 (22,798 – 66,007) | -- | -- | -- | -- | | -- | | -- | |
| Dec 2013 | 28,349 (18,126 – 54,405) | 0·99 | -- | -- | -- | | -- | | -- | |
| Upper River Region (URR) | |  |  |  |  |  | |  | |  |
| July 2013 | 63,387 (42,580 – 104,525) | -- | -- | -- | -- | | 0·93 | | -- | |
| Dec 2013 | 106,878 (53,245 – 203,160) | -- | 0·88 | -- | -- | | -- | | 0·21 | |
| Apr 2014 | 122,270 (90,475 – 182,254) | -- | -- | 0·97 | -- | | -- | | -- | |
| Dec 2014 | 96,051 (61,456 – 169,561) | -- | -- | -- | 0·96 | | -- | | -- | |
| Ages <15 years only | **AUC (95% CI)** | **p-value**  **July/Dec 2013 WCR** | **p-value**  **July/Dec 2013 URR** | **p-value**  **Dec 2013 / Apr 2014 URR** | **p-value**  **Apr/Dec 2014 URR** | | **p-value**  **WCR/URR July 2013** | | **p-value**  **WCR/URR Dec 2013** | |
| West Coast Region (WCR) | |  |  |  |  |  | |  | |  |
| July 2013 | 604 (486 – 838) | -- | -- | -- | -- | | -- | | -- | |
| Dec 2013 | 510 (430 – 635) | 0·996 | -- | -- | -- | | -- | | -- | |
| Upper River Region (URR) | |  |  |  |  |  | |  | |  |
| July 2013 | 1,322 (923 – 2,306) | -- | -- | -- | -- | | 0·56 | | -- | |
| Dec 2013 | 2,065 (1,564 – 2,897) | -- | 0·61 | -- | -- | | -- | | 0·02 | |
| Apr 2014 | 2,431 (2,024 – 3,065) | -- | -- | 0·98 | -- | | -- | | -- | |
| Dec 2014 | 2,429 (1,643 – 3,938) | -- | -- | -- | 0·87 | | -- | | -- | |

Supplementary Table 7. Rh2.2030 AUC values by geographical region and transmission season

| All ages | AUC (95% CI) | p-value  July/Dec 2013 WCR | p-value  July/Dec 2013 URR | p-value  Dec 2013 / Apr 2014 URR | p-value  Apr/Dec 2014 URR | | p-value  WCR/URR July 2013 | | p-value  WCR/URR Dec 2013 | |
| --- | --- | --- | --- | --- | --- | --- | --- | --- | --- | --- |
| West Coast Region (WCR) | |  |  |  |  |  | |  | |  |
| July 2013 | 70,330 (41,059 – 107,495) | -- | -- | -- | -- | | -- | | -- | |
| Dec 2013 | 42,208 (26,226 – 55,774) | 0·94 | -- | -- | -- | | -- | | -- | |
| Upper River Region (URR) | |  |  |  |  |  | |  | |  |
| July 2013 | 104,279 (61,392 – 183,017) | -- | -- | -- | -- | | 0·89 | | -- | |
| Dec 2013 | 227,633 (100,963 – 597,031) | -- | 0·72 | -- | -- | | -- | | 0·02 | |
| Apr 2014 | 170,801 (100,857 – 282,871) | -- | -- | 0·997 | -- | | -- | | -- | |
| Dec 2014 | 199,567 (151,882 – 365,945) | -- | -- | -- | 0·98 | | -- | | -- | |
| Ages <15 years only | **AUC (95% CI)** | **p-value**  **July/Dec 2013 WCR** | **p-value**  **July/Dec 2013 URR** | **p-value**  **Dec 2013 / Apr 2014 URR** | **p-value**  **Apr/Dec 2014 URR** | | **p-value**  **WCR/URR July 2013** | | **p-value**  **WCR/URR Dec 2013** | |
| West Coast Region (WCR) | |  |  |  |  |  | |  | |  |
| July 2013 | 2,540 (1,957 – 3,291) | -- | -- | -- | -- | | -- | | -- | |
| Dec 2013 | 1,629 (1,254 – 2,018) | 0·81 | -- | -- | -- | | -- | | -- | |
| Upper River Region (URR) | |  |  |  |  |  | |  | |  |
| July 2013 | 5,727 (3,675 – 8,722) | -- | -- | -- | -- | | 0·58 | | -- | |
| Dec 2013 | 14,198 (7,403 – 29,150) | -- | 0·32 | -- | -- | | -- | | <0·001 | |
| Apr 2014 | 10,225 (6,103 – 15,808) | -- | -- | 0·99 | -- | | -- | | -- | |
| Dec 2014 | 13,426 (9,254 – 26,133) | -- | -- | -- | 0·88 | | -- | | -- | |

Supplementary Table 8. *Pf*MSP1.19 AUC values by geographical region and transmission season

| All ages | AUC (95% CI) | p-value  July/Dec 2013 WCR | p-value  July/Dec 2013 URR | p-value  Dec 2013 / Apr 2014 URR | p-value  Apr/Dec 2014 URR | p-value  WCR/URR July 2013 | p-value  WCR/URR Dec 2013 |
| --- | --- | --- | --- | --- | --- | --- | --- |
| West Coast Region (WCR) | |  |  |  |  |  |  |
| July 2013 | 104,036 (79,703 – 137,412) | -- | -- | -- | -- | -- | -- |
| Dec 2013 | 79,069 (49,489 – 107,666) | 0·92 | -- | -- | -- | -- | -- |
| Upper River Region (URR) | |  |  |  |  |  |  |
| July 2013 | 133,067 (107,901 – 166,577) | -- | -- | -- | -- | 0·86 | -- |
| Dec 2013 | 217,626 (138,755 – 360,230) | -- | 0·37 | -- | -- | -- | 0·14 |
| Apr 2014 | 282,780 (223,343 – 360,160) | -- | -- | 1.00 | -- | -- | -- |
| Dec 2014 | 288,606 (223,580 – 384,970) | -- | -- | -- | 0·999 | -- | -- |
| Ages <15 years only | **AUC (95% CI)** | **p-value**  **July/Dec 2013 WCR** | **p-value**  **July/Dec 2013 URR** | **p-value**  **Dec 2013 / Apr 2014 URR** | **p-value**  **Apr/Dec 2014 URR** | **p-value**  **WCR/URR July 2013** | **p-value**  **WCR/URR Dec 2013** |
| West Coast Region (WCR) | |  |  |  |  |  |  |
| July 2013 | 6,198 (5,234 – 7,562) | -- | -- | -- | -- | -- | -- |
| Dec 2013 | 5,458 (4,469 – 7,481) | 0·97 | -- | -- | -- | -- | -- |
| Upper River Region (URR) | |  |  |  |  |  |  |
| July 2013 | 8,876 (7,689 – 10,289) | -- | -- | -- | -- | 0·79 | -- |
| Dec 2013 | 19,187 (14,281 – 26,016) | -- | 0·05 | -- | -- | -- | 0·02 |
| Apr 2014 | 21,399 (18,208 – 25,125) | -- | -- | 0·99 | -- | -- | -- |
| Dec 2014 | 22,640 (18,993 – 27,200) | -- | -- | -- | 0·99 | -- | -- |

Supplementary Table 9. *Pf*AMA1 AUC values by geographical region and transmission season

| All ages | AUC (95% CI) | p-value  July/Dec 2013 WCR | p-value  July/Dec 2013 URR | p-value  Dec 2013 / Apr 2014 URR | p-value  Apr/Dec 2014 URR | p-value  WCR/URR July 2013 | p-value  WCR/URR Dec 2013 |
| --- | --- | --- | --- | --- | --- | --- | --- |
| West Coast Region (WCR) | |  |  |  |  |  |  |
| July 2013 | 277,747 (183,416 – 391,223) | -- | -- | -- | -- | -- | -- |
| Dec 2013 | 223,176 (164,191 – 311,804) | 0·999 | -- | -- | -- | -- | -- |
| Upper River Region (URR) | |  |  |  |  |  |  |
| July 2013 | 420,297 (242,260 – 633,054) | -- | -- | -- | -- | 0·72 | -- |
| Dec 2013 | 546,793 (348,276 – 975,938) | -- | 0·93 | -- | -- | -- | 0·31 |
| Apr 2014 | 630,478 (332,041 – 960,525) | -- | -- | 0·99 | -- | -- | -- |
| Dec 2014 | 664550 (488,529 – 1,158,277) | -- | -- | -- | 0·97 | -- | -- |
| Ages <15 years only | **AUC (95% CI)** | **p-value**  **July/Dec 2013 WCR** | **p-value**  **July/Dec 2013 URR** | **p-value**  **Dec 2013 / Apr 2014 URR** | **p-value**  **Apr/Dec 2014 URR** | **p-value**  **WCR/URR July 2013** | **p-value**  **WCR/URR Dec 2013** |
| West Coast Region (WCR) | |  |  |  |  |  |  |
| July 2013 | 2,932 (2,119 – 3,805) | -- | -- | -- | -- | -- | -- |
| Dec 2013 | 2,420 (1,837 – 3,206) | 0·998 | -- | -- | -- | -- | -- |
| Upper River Region (URR) | |  |  |  |  |  |  |
| July 2013 | 17,434 (9,952 – 24,963) | -- | -- | -- | -- | 0·08 | -- |
| Dec 2013 | 28,679 (18,302 – 50,513) | -- | 0·62 | -- | -- | -- | <0·001 |
| Apr 2014 | 22,561 (12,614 – 30,647) | -- | -- | 0·95 | -- | -- | -- |
| Dec 2014 | 28,572 (20,057 – 54,152) | -- | -- | -- | 0·84 | -- | -- |

Supplementary Table 10. *Pf*GLURP.R2 AUC values by geographical region and transmission season

| All ages | AUC (95% CI) | p-value  July/Dec 2013 WCR | p-value  July/Dec 2013 URR | p-value  Dec 2013 / Apr 2014 URR | p-value  Apr/Dec 2014 URR | p-value  WCR/URR July 2013 | p-value  WCR/URR Dec 2013 |
| --- | --- | --- | --- | --- | --- | --- | --- |
| West Coast Region (WCR) | |  |  |  |  |  |  |
| July 2013 | 152,583 (101,298 – 245,028) | -- | -- | -- | -- | -- | -- |
| Dec 2013 | 137,373 (75,927 – 236,820) | 0.97 | -- | -- | -- | -- | -- |
| Upper River Region (URR) | |  |  |  |  |  |  |
| July 2013 | 216,273 (140,606 – 329,051) | -- | -- | -- | -- | 0.84 | -- |
| Dec 2013 | 535,952 (275,903 – 931,804) | -- | 0.55 | -- | -- | -- | 0.09 |
| Apr 2014 | 516,108 (350,947 – 851,814) | -- | -- | 0.96 | -- | -- | -- |
| Dec 2014 | 507,106 (380,953 – 693,642) | -- | -- | -- | 1.00 | -- | -- |
| Ages <15 years only | **AUC (95% CI)** | **p-value**  **July/Dec 2013 WCR** | **p-value**  **July/Dec 2013 URR** | **p-value**  **Dec 2013 / Apr 2014 URR** | **p-value**  **Apr/Dec 2014 URR** | **p-value**  **WCR/URR July 2013** | **p-value**  **WCR/URR Dec 2013** |
| West Coast Region (WCR) | |  |  |  |  |  |  |
| July 2013 | 945 (716 – 1,330) | -- | -- | -- | -- | -- | -- |
| Dec 2013 | 1,332 (1,050 – 1,771) | 0.98 | -- | -- | -- | -- | -- |
| Upper River Region (URR) | |  |  |  |  |  |  |
| July 2013 | 4,438 (2,910 – 6,471) | -- | -- | -- | -- | 0.35 | -- |
| Dec 2013 | 5,890 (4,367 – 7,980) | -- | 0.56 | -- | -- | -- | 0.04 |
| Apr 2014 | 6,583 (4,982 – 9,306) | -- | -- | 0.88 | -- | -- | -- |
| Dec 2014 | 6,672 (5,127 – 8,392) | -- | -- | -- | 0.999 | -- | -- |

Supplementary Table 11. Etramp5.Ag1 antibody responses during dry season prior to MDA in Upper River Region and odds of clinical malaria or asymptomatic *P.falciparum* infection during transmission season after MDA. Clinical malaria cases are detected passively at the health facility or in the community by study nurses. Asymptomatic *P.falciparum* infections are detected by PCR from monthly survey. Odds ratios are shown unadjusted and adjusted for age, LLIN use and MDA compliance.

| Outcome: Clinical malaria (passive case detection or fever and RDT-positive) | | | | | | |
| --- | --- | --- | --- | --- | --- | --- |
|  | **Unadjusted** | | | **Adjusted** | | |
|  | **OR** | **95%CI** | **p-value** | **aOR** | **95%CI** | **p-value** |
| Above age-average MFI (pre-MDA) | | | | | | |
| Lower 50^th^ percentile | 1·54 | 0·87 – 2·72 | 0·14 | 1·23 | 0·64 – 2·35 | 0·55 |
| Upper 50^th^ percentile | 1·84 | 1·06 – 3·20 | 0·03 | 2·05 | 1·12 – 3·77 | 0·02 |
| Age | -- | -- | -- | 0·99 | 0·98 – 1·01 | 0·52 |
| LLIN use 24 hours | -- | -- | -- | 0·95 | 0·88 – 1·02 | 0·16 |
| MDA compliance | -- | -- | -- | 0·98 | 0·87 – 1·10 | 0·76 |

| Outcome: Asymptomatic *P.falciparum* infection (detected by PCR) | | | | | | |
| --- | --- | --- | --- | --- | --- | --- |
|  | **Unadjusted** | | | **Adjusted** | | |
|  | **OR** | **95%CI** | **p-value** | **aOR** | **95%CI** | **p-value** |
| Above age-average MFI (pre-MDA) | | | | | | |
| Lower 50^th^ percentile | 1·32 | 0·84 – 2·05 | 0·23 | 0·96 | 0·58 – 1·64 | 0·89 |
| Upper 50^th^ percentile | 1·31 | 0·84 – 2·04 | 0·24 | 1·14 | 0·68 – 1·91 | 0·63 |
| Age | -- | -- | -- | 0·99 | 0·98 – 1·01 | 0·26 |
| LLIN use 24 hours | -- | -- | -- | 0·97 | 0·91 – 1·03 | 0·29 |
| MDA compliance | -- | -- | -- | 1·00 | 0·91 – 1·09 | 0·93 |

Supplementary Table 12. GEXP18 antibody responses during dry season prior to MDA in Upper River Region and odds of clinical malaria or asymptomatic *P.falciparum* infection during transmission season after MDA. Clinical malaria cases are detected passively at the health facility or in the community by study nurses. Asymptomatic *P.falciparum* infections are detected by PCR from monthly survey. Odds ratios are shown unadjusted and adjusted for age, LLIN use and MDA compliance.

| Outcome: Clinical malaria (passive case detection or fever and RDT-positive) | | | | | | |
| --- | --- | --- | --- | --- | --- | --- |
|  | **Unadjusted** | | | **Adjusted** | | |
|  | **OR** | **95%CI** | **p-value** | **aOR** | **95%CI** | **p-value** |
| Above age-average MFI (pre-MDA) | | | | | | |
| Lower 50^th^ percentile | 1·19 | 0·66 – 2·14 | 0·56 | 1·03 | 0·53 – 1·99 | 0·93 |
| Upper 50^th^ percentile | 1·48 | 0·85 – 2·59 | 0·17 | 1·49 | 0·80 – 2·75 | 0·21 |
| Age | -- | -- | -- | 1·00 | 0·98 – 1·01 | 0·74 |
| LLIN use 24 hours | -- | -- | -- | 0·95 | 0·88 – 1·02 | 0·13 |
| MDA compliance | -- | -- | -- | 0·98 | 0·87 – 1·10 | 0·75 |

| Outcome: Asymptomatic *P.falciparum* infection (detected by PCR) | | | | | | |
| --- | --- | --- | --- | --- | --- | --- |
|  | **Unadjusted** | | | **Adjusted** | | |
|  | **OR** | **95%CI** | **p-value** | **aOR** | **95%CI** | **p-value** |
| Above age-average MFI (pre-MDA) | | | | | | |
| Lower 50^th^ percentile | 0·88 | 0·47 – 1·65 | 0·69 | 0·76 | 0·37 – 1·54 | 0·44 |
| Upper 50^th^ percentile | 0·71 | 0·36 – 1·42 | 0·33 | 0·54 | 0·24 – 1·22 | 0·14 |
| Age | -- | -- | -- | 0·99 | 0·97 – 1·01 | 0·25 |
| LLIN use 24 hours | -- | -- | -- | 0·97 | 0·89 – 1·07 | 0·56 |
| MDA compliance | -- | -- | -- | 0·97 | 0·85 – 1·11 | 0·70 |

Supplementary Table 13. HSP40.Ag1 antibody responses during dry season prior to MDA in Upper River Region and odds of clinical malaria or asymptomatic *P.falciparum* infection during transmission season after MDA. Clinical malaria cases are detected passively at the health facility or in the community by study nurses. Asymptomatic *P.falciparum* infections are detected by PCR from monthly survey. Odds ratios are shown unadjusted and adjusted for age, LLIN use and MDA compliance.

| Outcome: Clinical malaria (passive case detection or fever and RDT-positive) | | | | | | |
| --- | --- | --- | --- | --- | --- | --- |
|  | **Unadjusted** | | | **Adjusted** | | |
|  | **OR** | **95%CI** | **p-value** | **aOR** | **95%CI** | **p-value** |
| Above age-average MFI (pre-MDA) | | | | | | |
| Lower 50^th^ percentile | 0·94 | 0·51 – 1·72 | 0·84 | 0·85 | 0·43 – 1·66 | 0·63 |
| Upper 50^th^ percentile | 1·51 | 0·87 – 2·62 | 0·14 | 1·54 | 0·83 – 2·84 | 0·17 |
| Age | -- | -- | -- | 1·00 | 0·98 – 1·01 | 0·63 |
| LLIN use 24 hours* | -- | -- | -- | -- | -- | -- |
| MDA compliance | -- | -- | -- | 1·00 | 0·88 – 1·12 | 0·94 |

*Not adjusted for LLIN use due to failure for model to converge

| Outcome: Asymptomatic *P.falciparum* infection (detected by PCR) | | | | | | |
| --- | --- | --- | --- | --- | --- | --- |
|  | **Unadjusted** | | | **Adjusted** | | |
|  | **OR** | **95%CI** | **p-value** | **aOR** | **95%CI** | **p-value** |
| Above age-average MFI (pre-MDA) | | | | | | |
| Lower 50^th^ percentile | 1·77 | 0·95 – 3·29 | 0·07 | 1·64 | 0·81 – 3·34 | 0·17 |
| Upper 50^th^ percentile | 0·98 | 0·48 – 1·99 | 0·95 | 0·75 | 0·32 – 1·77 | 0·52 |
| Age | -- | -- | -- | 0·99 | 0·97 – 1·01 | 0·43 |
| LLIN use 24 hours | -- | -- | -- | 0·97 | 0·89 – 1·06 | 0·52 |
| MDA compliance | -- | -- | -- | 0·97 | 0·86 – 1·11 | 0·70 |

Supplementary Table 14. EBA175 antibody responses during dry season prior to MDA in Upper River Region and odds of clinical malaria or asymptomatic *P.falciparum* infection during transmission season after MDA. Clinical malaria cases are detected passively at the health facility or in the community by study nurses. Asymptomatic *P.falciparum* infections are detected by PCR from monthly survey. Odds ratios are shown unadjusted and adjusted for age, LLIN use and MDA compliance.

| Outcome: Clinical malaria (passive case detection or fever and RDT-positive) | | | | | | |
| --- | --- | --- | --- | --- | --- | --- |
|  | **Unadjusted** | | | **Adjusted** | | |
|  | **OR** | **95%CI** | **p-value** | **aOR** | **95%CI** | **p-value** |
| Above age-average MFI (pre-MDA) | | | | | | |
| Lower 50^th^ percentile | 1·69 | 1·00 – 2·85 | 0·05 | 1·64 | 0·91 – 2·93 | 0·10 |
| Upper 50^th^ percentile | 1·03 | 0·55 – 1·94 | 0·92 | 1·03 | 0·49 – 2·16 | 0·94 |
| Age | -- | -- | -- | 1·00 | 0·98 – 1·02 | 0·99 |
| LLIN use 24 hours | -- | -- | -- | 0·94 | 0·88 – 1·02 | 0·13 |
| MDA compliance | -- | -- | -- | 0·98 | 0·87 – 1·10 | 0·71 |

| Outcome: Asymptomatic *P.falciparum* infection (detected by PCR) | | | | | | |
| --- | --- | --- | --- | --- | --- | --- |
|  | **Unadjusted** | | | **Adjusted** | | |
|  | **OR** | **95%CI** | **p-value** | **aOR** | **95%CI** | **p-value** |
| Above age-average MFI (pre-MDA) | | | | | | |
| Lower 50^th^ percentile | 1·23 | 0·66 – 2·29 | 0·52 | 1·13 | 0·57 – 2·26 | 0·73 |
| Upper 50^th^ percentile | 0·70 | 0·32 – 1·53 | 0·37 | 0·55 | 0·20 – 1·53 | 0·25 |
| Age | -- | -- | -- | 0·99 | 0·97 – 1·01 | 0·51 |
| LLIN use 24 hours | -- | -- | -- | 0·97 | 0·89 – 1·06 | 0·54 |
| MDA compliance | -- | -- | -- | 0·97 | 0·85 – 1·10 | 0·61 |

Supplementary Table 15. Rh2.2030 antibody responses during dry season prior to MDA in Upper River Region and odds of clinical malaria or asymptomatic *P.falciparum* infection during transmission season after MDA. Clinical malaria cases are detected passively at the health facility or in the community by study nurses. Asymptomatic *P.falciparum* infections are detected by PCR from monthly survey. Odds ratios are shown unadjusted and adjusted for age, LLIN use and MDA compliance.

| Outcome: Clinical malaria (passive case detection or fever and RDT-positive) | | | | | | |
| --- | --- | --- | --- | --- | --- | --- |
|  | **Unadjusted** | | | **Adjusted** | | |
|  | **OR** | **95%CI** | **p-value** | **aOR** | **95%CI** | **p-value** |
| Above age-average MFI (pre-MDA) | | | | | | |
| Lower 50^th^ percentile | 1·49 | 0·84 – 2·63 | 0·17 | 1·41 | 0·75 – 2·64 | 0·28 |
| Upper 50^th^ percentile | 2·09 | 1·21 – 3·62 | 0·01 | 2·13 | 1·14 – 3·99 | 0·02 |
| Age | -- | -- | -- | 0·99 | 0·98 – 1·01 | 0·56 |
| LLIN use 24 hours | -- | -- | -- | 0·95 | 0·88 – 1·02 | 0·13 |
| MDA compliance | -- | -- | -- | 0·99 | 0·88 – 1·11 | 0·88 |

| Outcome: Asymptomatic *P.falciparum* infection (detected by PCR) | | | | | | |
| --- | --- | --- | --- | --- | --- | --- |
|  | **Unadjusted** | | | **Adjusted** | | |
|  | **OR** | **95%CI** | **p-value** | **aOR** | **95%CI** | **p-value** |
| Above age-average MFI (pre-MDA) | | | | | | |
| Lower 50^th^ percentile | 1·56 | 0·83 – 2·96 | 0·17 | 1·60 | 0·81 – 3·14 | 0·17 |
| Upper 50^th^ percentile | 1·52 | 0·79 – 2·95 | 0·21 | 1·27 | 0·57 – 2·85 | 0·55 |
| Age | -- | -- | -- | 0·99 | 0·97 – 1·01 | 0·26 |
| LLIN use 24 hours* | -- | -- | -- | -- | -- | -- |
| MDA compliance | -- | -- | -- | 0·98 | 0·86 – 1·11 | 0·70 |

*Not adjusted for LLIN use due to failure for model to converge

Supplementary Table 16. *Pf*MSP1.19 antibody responses during dry season prior to MDA in Upper River Region and odds of clinical malaria or asymptomatic *P.falciparum* infection during transmission season after MDA. Clinical malaria cases are detected passively at the health facility or in the community by study nurses. Asymptomatic *P.falciparum* infections are detected by PCR from monthly survey. Odds ratios are shown unadjusted and adjusted for age, LLIN use and MDA compliance.

| Outcome: Clinical malaria (passive case detection or fever and RDT-positive) | | | | | | |
| --- | --- | --- | --- | --- | --- | --- |
|  | **Unadjusted** | | | **Adjusted** | | |
|  | **OR** | **95%CI** | **p-value** | **aOR** | **95%CI** | **p-value** |
| Above age-average MFI (pre-MDA) | | | | | | |
| Lower 50^th^ percentile | 0·83 | 0·46 – 1·49 | 0·53 | 0·71 | 0·37 – 1·35 | 0·30 |
| Upper 50^th^ percentile | 1·22 | 0·71 – 2·11 | 0·46 | 1·06 | 0·56 – 1·99 | 0·86 |
| Age | -- | -- | -- | 1·00 | 0·98 – 1·01 | 0·69 |
| LLIN use 24 hours | -- | -- | -- | 0·94 | 0·88 – 1·01 | 0·11 |
| MDA compliance | -- | -- | -- | 0·98 | 0·87 – 1·10 | 0·75 |

| Outcome: Asymptomatic *P.falciparum* infection (detected by PCR) | | | | | | |
| --- | --- | --- | --- | --- | --- | --- |
|  | **Unadjusted** | | | **Adjusted** | | |
|  | **OR** | **95%CI** | **p-value** | **aOR** | **95%CI** | **p-value** |
| Above age-average MFI (pre-MDA) | | | | | | |
| Lower 50^th^ percentile | 1·17 | 0·64 – 2·13 | 0·61 | 1·22 | 0·63 – 2·34 | 0·56 |
| Upper 50^th^ percentile | 0·55 | 0·25 – 1·21 | 0·14 | 0·69 | 0·29 – 1·67 | 0·41 |
| Age | -- | -- | -- | 0·99 | 0·97 – 1·01 | 0·36 |
| LLIN use 24 hours | -- | -- | -- | 0·97 | 0·89 – 1·07 | 0·58 |
| MDA compliance | -- | -- | -- | 0·97 | 0·85 – 1·11 | 0·66 |

Supplementary Table 17. *Pf*AMA1 antibody responses during dry season prior to MDA in Upper River Region and odds of clinical malaria or asymptomatic *P.falciparum* infection during transmission season after MDA. Clinical malaria cases are detected passively at the health facility or in the community by study nurses. Asymptomatic *P.falciparum* infections are detected by PCR from monthly survey. Odds ratios are shown unadjusted and adjusted for age, LLIN use and MDA compliance.

| Outcome: Clinical malaria (passive case detection or fever and RDT-positive) | | | | | | |
| --- | --- | --- | --- | --- | --- | --- |
|  | **Unadjusted** | | | **Adjusted** | | |
|  | **OR** | **95%CI** | **p-value** | **aOR** | **95%CI** | **p-value** |
| Above age-average MFI (pre-MDA) | | | | | | |
| Lower 50^th^ percentile | 1·51 | 0·86 – 2·66 | 0·15 | 1·68 | 0·91 – 3·10 | 0·10 |
| Upper 50^th^ percentile | 1·71 | 0·98 – 3·01 | 0·06 | 1·63 | 0·85 – 3·13 | 0·14 |
| Age | -- | -- | -- | 1·00 | 0·98 – 1·01 | 0·65 |
| LLIN use 24 hours | -- | -- | -- | 0·95 | 0·88 – 1·02 | 0·18 |
| MDA compliance | -- | -- | -- | 0·99 | 0·88 – 1·11 | 0·82 |

| Outcome: Asymptomatic *P.falciparum* infection (detected by PCR) | | | | | | |
| --- | --- | --- | --- | --- | --- | --- |
|  | **Unadjusted** | | | **Adjusted** | | |
|  | **OR** | **95%CI** | **p-value** | **aOR** | **95%CI** | **p-value** |
| Above age-average MFI (pre-MDA) | | | | | | |
| Lower 50^th^ percentile | 0·87 | 0·44 – 1·72 | 0·95 | 0·74 | 0·35 – 1·57 | 0·43 |
| Upper 50^th^ percentile | 1·08 | 0·57 – 2·04 | 0·80 | 0·97 | 0·45 – 2·09 | 0·95 |
| Age | -- | -- | -- | 0·99 | 0·97 – 1·01 | 0·26 |
| LLIN use 24 hours | -- | -- | -- | 0·97 | 0·89 – 1·06 | 0·53 |
| MDA compliance | -- | -- | -- | 0·97 | 0·85 – 1·11 | 0·69 |

Supplementary Table 18. *Pf*GLURP.R2 antibody responses during dry season prior to MDA in Upper River Region and odds of clinical malaria or asymptomatic *P.falciparum* infection during transmission season after MDA. Clinical malaria cases are detected passively at the health facility or in the community by study nurses. Asymptomatic *P.falciparum* infections are detected by PCR from monthly survey. Odds ratios are shown unadjusted and adjusted for age, LLIN use and MDA compliance.

| Outcome: Clinical malaria (passive case detection or fever and RDT-positive) | | | | | | |
| --- | --- | --- | --- | --- | --- | --- |
|  | **Unadjusted** | | | **Adjusted** | | |
|  | **OR** | **95%CI** | **p-value** | **aOR** | **95%CI** | **p-value** |
| Above age-average MFI (pre-MDA) | | | | | | |
| Lower 50^th^ percentile | 1·06 | 0·59 – 1·89 | 0·85 | 1·15 | 0·62 – 2·14 | 0·65 |
| Upper 50^th^ percentile | 1·43 | 0·83 – 2·47 | 0·20 | 1·19 | 0·59 – 2·39 | 0·63 |
| Age | -- | -- | -- | 1·00 | 0·98 – 1·01 | 0·66 |
| LLIN use 24 hours | -- | -- | -- | 0·95 | 0·88 – 1·02 | 0·15 |
| MDA compliance | -- | -- | -- | 0·98 | 0·87 – 1·11 | 0·78 |

| Outcome: Asymptomatic *P.falciparum* infection (detected by PCR) | | | | | | |
| --- | --- | --- | --- | --- | --- | --- |
|  | **Unadjusted** | | | **Adjusted** | | |
|  | **OR** | **95%CI** | **p-value** | **aOR** | **95%CI** | **p-value** |
| Above age-average MFI (pre-MDA) | | | | | | |
| Lower 50^th^ percentile | 0·86 | 0·44 – 1·69 | 0·67 | 0·91 | 0·45 – 1·85 | 0·80 |
| Upper 50^th^ percentile | 0·74 | 0·36 – 1·52 | 0·41 | 0·59 | 0·23 – 1·54 | 0·28 |
| Age | -- | -- | -- | 0·99 | 0·97 – 1·01 | 0·50 |
| LLIN use 24 hours | -- | -- | -- | 0·97 | 0·89 – 1·06 | 0·52 |
| MDA compliance | -- | -- | -- | 0·97 | 0·85 – 1·11 | 0·67 |
